# The Effectiveness of External Verbal Feedback on Balance in Athletes with Chronic Ankle Instability

**DOI:** 10.3390/jfmk9010056

**Published:** 2024-03-21

**Authors:** Konstantinos Parlakidis, Lazaros Alexandors Kontopoulos, Dimitris Mandalidis, Eleftherios Paraskevopoulos, Maria Papandreou, Eleni Kapreli, Anna Christakou

**Affiliations:** 1 Department of Physiotherapy, University of Peloponnese, 23100 Sparta, Greece; konstantinosparlakidis1@gmail.com (K.P.); alexkont4@gmail.com (L.A.K.); 2 Department of Physical Education and Sports Science, University of Athens, 11527 Athens, Greece; dmndldis@phed.uoa.gr; 3 Laboratory of Advanced Physiotherapy, Department of Physiotherapy, University of West Attica, 23100 Sparta, Greece; elparaskevop@uniwa.gr (E.P.); mpapand@uniwa.gr (M.P.); 4 Laboratory of Biomechanics, Department of Physiotherapy, University of Peloponnese, 23100 Sparta, Greece; 5 Department of Physiotherapy, Clinical Exercise Physiology and Rehabilitation Research Laboratory, University of Thessaly, 41500 Larissa, Greece; ekapreli@uth.gr

**Keywords:** chronic instability, ankle, additional feedback, balance

## Abstract

Chronic ankle instability (CAI) is characterized by muscle weakness and impaired neuromuscular control. This study aimed (a) to assess the impact of external verbal feedback on the dynamic balance of athletes with CAI and (b) to examine the maintenance of dynamic balance ability after the end of the completion of the intervention balance program. Thirty athletes (mean age 21.63 ± 1.53) were randomly divided into three groups: an experimental group with external verbal feedback, 1st control group without external verbal feedback and the 2nd control group without balance training and without feedback. Assessments using a balance board and the ‘Y-balance’ test were conducted before and after the balance training period. Additionally, participants completed the Cumberland Ankle Joint Instability Tool. A retention test of balance ability was administered after the 4-week intervention period. Statistical analysis revealed a significant overall improvement in balance (F(2,36) =5.96, *p* = 0.006, partial η^2^ =0.249), including those with no balance training, but no significant differences between the groups. Thus, the external verbal feedback did not show a positive impact on the balance ability between the three different groups. Also, the experimental group with the external verbal feedback demonstrated maintenance of dynamic balance learning ability. Although it appears that balance training has a positive effect on the dynamic balance of individuals with CAI, a non-positive impact of external verbal feedback was found. Also, it appears that external verbal feedback significantly led to sustained retention of balance learning ability. Further research is recommended to validate these findings.

## 1. Introduction

Ankle sprains represent a prevalent musculoskeletal injury in sports, affecting athletes across various disciplines such as basketball (41.1%), rugby (9.3%), and football (7.9%) [1]. Individuals who sustain ankle sprains often exhibit a range of impairments including decreased proprioception, delayed neuromuscular control, pain, and susceptibility to re-injury, which can lead to the development of chronic ankle instability (CAI) [2,3]. CAI patients commonly experience recurrent or excessive sprains, affecting approximately 15.7% of individuals with ankle injuries [4,5]. Symptoms associated with CAI include pain, swelling, muscle weakness, instability, reduced proprioception, limited range of motion, and delayed neuromuscular control [6,7,8,9]. These symptoms can significantly impact an athlete’s performance and increase the risk of further injury, highlighting the importance of effective management and rehabilitation strategies for individuals with CAI.

Balance plays a crucial role in the rehabilitation of CAI, as it is recognized as a key factor influencing the risk of recurrent sprains [10]. Numerous studies have demonstrated significant improvements in both static [11,12,13] and dynamic balance [11,12,14] among individuals with CAI following participation in physiotherapy rehabilitation programs. Specifically, balance retraining has been shown to activate the somatosensory system [15] and enhance participants’ strength and functional ability [9,16]. These findings underscore the importance of incorporating balance training into CAI rehabilitation protocols to address deficits in proprioception, stability, and neuromuscular control, thereby reducing the risk of future ankle injuries.

Motor learning refers to an individual’s ability to acquire motor skills [17], which has broad applications in enhancing sports performance [18,19], preventing injuries [20,21], and facilitating rehabilitation [22,23]. Within the domain of motor learning, attentional focus, observational learning, and feedback are key components that are widely utilized in sports [24,25]. Feedback, in particular, plays a critical role in providing information to individuals about the outcome or process of their actions [26]. Feedback can be categorized as internal (intrinsic) or external (extrinsic), with external feedback further divided into verbal, motor, and feedback delivered via taped videos [27]. Research has shown that feedback is effective in improving performance in both healthy populations [28,29,30,31,32] and clinical populations undergoing rehabilitation [22,33,34]. By providing individuals with information about their performance, feedback allows for adjustments and improvements in motor skills, contributing to enhanced athletic performance and rehabilitation outcomes.

The existing research on the impact of feedback on the balance of musculoskeletal injured athletes is limited, with only one previous study focusing on the effects of verbal feedback on individuals with chronic ankle instability (CAI) [33]. Furthermore, there is a gap in the literature regarding the maintenance of dynamic balance ability following the completion of a balance program and external verbal feedback intervention. Additionally, there is a lack of experimental research investigating the psychophysiological processes involved in applying external verbal feedback in sports injury rehabilitation.

Understanding the psychophysiological processes underlying sports injury rehabilitation is not only theoretically important but also holds clinical significance. Such knowledge could lead to the development of more effective rehabilitation programs aimed at improving sports performance. Therefore, the primary objective of this study was to investigate the impact of external verbal feedback on the dynamic balance of athletes with CAI. It was hypothesized that external verbal feedback would lead to improvements in dynamic balance among patients with CAI and that these improvements would be sustained even after the completion of the feedback intervention.

## 2. Materials and Methods

The present study is a randomized single blind study.

### 2.1. Participants

All participants were provided with detailed information regarding the study procedures and were required to sign a written consent form before participating. They were informed of their right to withdraw from the study at any point, and assured that any publication of the results would maintain their anonymity. The sample comprised 30 athletes, consisting of 18 men and 12 women, with ages ranging from 18 to 30 years old (mean age = 21.63 years, standard deviation = 1.53 years). All participants had a minimum of two years of experience participating in sports competitions. They were diagnosed with chronic ankle instability (CAI) affecting either the left or right ankle joint (see Table 1).

The participants were randomly assigned to one of three groups using a drawing lots method: (a) an experimental group that received balance training along with external verbal feedback, (b) the 1st control group that underwent balance training without external verbal feedback, and (c) the 2nd control group that did not receive any balance training or external verbal feedback. Each group comprised 10 participants. In the experimental group, there were 3 men (30%) and 7 women (70%), in the 1st control group there were 6 men (60%) and 4 women (40%), and in the 2nd control group, there were 9 men (90%) and 1 woman (10%).

To be eligible for inclusion in the study, participants had to meet the following criteria: (i) diagnosis of CAI, (ii) aged between 18 and 40 years old, (iii) undergone conservative medical care in either a public hospital or private clinic, and (iv) engaged in sports activities at least three times weekly over the past two years. Exclusion criteria included: (i) visual disorders, (ii) vestibular or neurological disorders, (iii) history of surgery on the ankle joint or adjacent joints (e.g., knee), (iv) no fractures, (v) no severe traumas on the lower limbs, and (vi) no ankle sprains in the last 3 months or participation in physiotherapy programs in the last 3 months. The study was approved by the Ethics Committee of the University of Peloponnese (6 April 2023, No 206).

### 2.2. Measures

**Figure 1:**
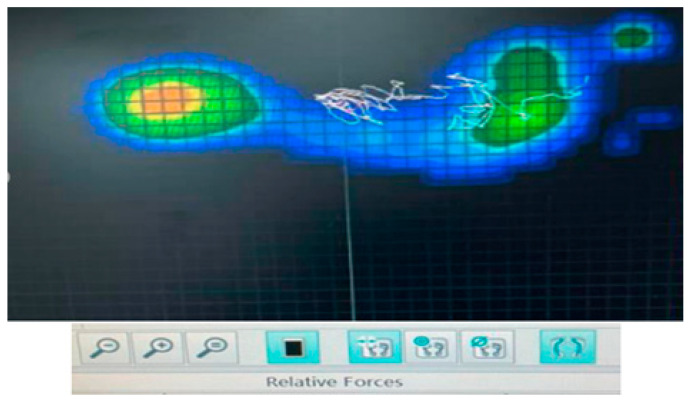
Footprint with the distribution of pressures.

The balance assessment instruments and tests utilized in our study were validated, reliable, and widely employed [35,36,37,38,39,40,41,42,43,44,45]. Specifically, we employed:

(a) Y-Balance test on a plantar pressure recording platform:

The Y-Balance test [35] was utilized to evaluate dynamic balance in individuals with chronic ankle instability [36]. The plantar pressure recording platform comprised 1792 capacitive force sensors arranged in a 32 cm × 56 cm matrix, synchronized with a personal computer. Foot pressure signals were recorded at a sampling rate of 120 Hz and analyzed using dedicated computer software (WinFDMS v.1.18.40, Zebris Medical GmbH Standort Seltmans, Weitnau, Germany) [37]. The recorded images displayed the footprint of each participant’s foot, with hot colors (red, orange, yellow) indicating higher pressures and cold colors (blue, green) indicating lower pressures [38] (Figure 1). This pressure distribution platform has been previously used by Cobos-Moreno et al. [39] in a study involving 52 subjects to identify physiological lower limb pressures, demonstrating a high reliability index (r = 0.95) [39]. Additionally, Mandalidis and Karagiannakis [40] employed it to assess postural control during a dynamic balance test, showing high reliability (ICC = 0.87). Center of Pressure (CoP) path length and CoP path velocity are indices derived from the platform, with the former reflecting values related to the oscillation of the center during the test and the latter indicating the speed at which the person moves during the test [40]. Therefore, we examined both the center of pressure distribution length and the center of pressure distribution velocity.

(b) Balance tray

The balance tray is a circular platform equipped with various-sized domes that protrude from the bottom of the tray, allowing for adjustments to the difficulty level of balance tasks [11]. This equipment, known as a balance disc, is widely recognized as a valid and reliable instrument for balance retraining and has been utilized in interventions for individuals with chronic ankle instability [41,42,43].

(c) Cumberland Ankle Joint Instability Tool (CAIT)

The Cumberland Ankle Instability Tool (CAIT) is a widely used assessment tool for selecting individuals with ankle instability. It consists of 9 items designed to evaluate ankle joint instability during various functional activities. The score of the tool ranges from 30-0, where a score of ≤24 indicates chronic ankle instability in individuals who have experienced sprains. The CAIT has demonstrated high validity and reliability, with a Cronbach’s alpha coefficient of 0.83 [44,45].

### 2.3. Procedure

The participants were recruited by the authors through personal or telephone contact, or through their coach. During the first meeting, they completed the Cumberland Ankle Instability Tool (CAIT) to be assessed for eligibility by the last author. Thirty-six athletes met the inclusion criteria, but only 30 agreed to participate and completed the demographic data sheet. In the second meeting, the participants were randomly assigned into three groups using the method of drawing lots, conducted by the fourth author.

All participants’ balance ability was evaluated by the second author, who was unaware of each participant’s randomization group. The first assessment of dynamic balance was performed using the Y-Balance test on a pressure distribution platform, specifically in the forward direction only. This direction was chosen as it has a larger area and requires less speed compared to the rearward directions [46]. The length and velocity of the center of pressure distribution were measured during the Y-balance test using a plantar pressure recording platform.

**Figure 2:**
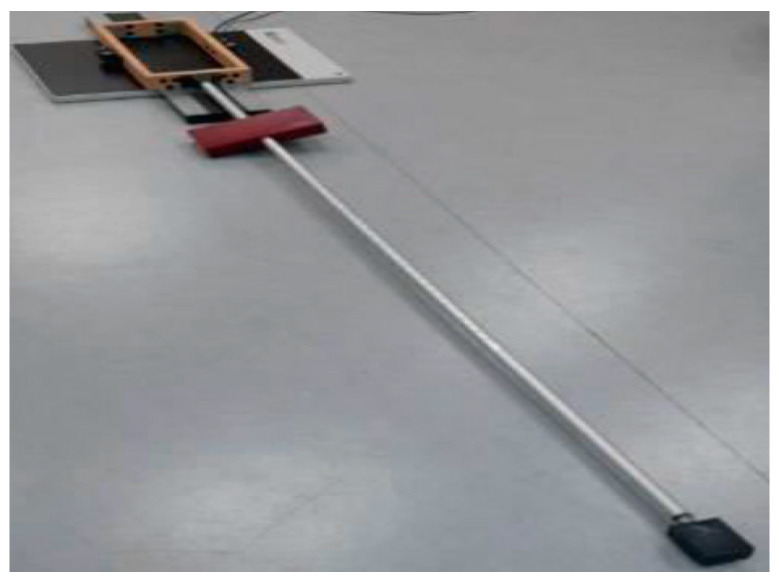
Y-Balance test on a plantar pressure recording platform.

During the Y-Balance test on the pressure distribution platform, participants performed reaches as far as possible with one lower limb anteriorly, while standing at the other end with their hands on the iliac spines. Each participant completed three trial attempts and three normal attempts on the Y-balance test, with a one-minute rest between repetitions to prevent fatigue. The mean values of the three normal attempts were used to calculate the score. The score for each direction was calculated by dividing the average distance reached by the length of the participant’s lower limb, then multiplying by 100 to obtain the percentage of lower limb length. The length of the lower limb was measured from the anterior superior iliac spine to the medial malleolus. To calculate the score, the sum of the attempts was divided by three times the limb length (Figure 2).

All participants completed the CAIT prior to (a) the 1st assessment of dynamic balance, (b) the 2nd assessment of dynamic balance (4th week), and (c) the retention of the learning phase of dynamic ability (the 3rd assessment) (8th week). In the experimental group, participants engaged in a balance training program with both lower limbs on a balance tray, receiving specific verbal feedback from the first author. The training frequency was three sessions per week for duration of four weeks [47]. Each training session consisted of seven trials, each lasting 90 s, with breaks between each trial to avoid fatigue [48]. Participants wore their preferred training shoes during the sessions. Verbal feedback instructions given to the experimental group included: “Try to keep the tray steady” or “Try to keep the tray in a straight line”, “Straighten the back”, “Lower the center of gravity”, “Bend (the) knees”, “Head forward and eyes looking straight ahead”, “Don’t hold your breath”, “Stretch your arms out to the side” as appropriate for each participant. Participants were instructed to step onto the balance board using their left foot first and then put their right one on while keeping their weight on the left, ensuring that the platform touched the ground on the left side. They waited for the examiner’s signal to start balancing by distributing weight on both legs. At the end of each trial, participants were instructed to stop balancing on the board by putting all their weight on the left side and stepping off the board using their right foot first.

The 1st control group followed the same balance program without external verbal feedback, receiving only internal feedback. The 2nd control group did not follow any balance program or receive external verbal feedback. At the end of the intervention program, a 2nd assessment of dynamic balance was conducted for the entire sample by the second author. After four weeks, a 3rd assessment of dynamic balance was conducted for the entire sample, serving as a retention evaluation assessment of balance ability.

### 2.4. Statistical Analyses

Descriptive statistics were used to examine the demographic data of the sample.. Homogeneity of sample demographics was assessed using independent samples *t*-tests with a significance level of α = 0.05. A normality test of the distribution of all variables was conducted using the Shapiro-Wilk normality test. Repeated measures ANOVA (Mixed ANOVA) was performed on the variable of balance ability, including (a) Y-balance test, (b) center length and center velocity pressure distribution, and (c) CAI test. Greenhouse-Geisser correction was applied when necessary. Kruskal-Wallis non-parametric H test comparisons were used between the three groups to compare the 1st and the 3rd measurement and the 2nd and the 3rd measurement on center length and center velocity pressure distribution during the Y-balance test. Separate paired *t*-test comparisons of repeated measures in each group on the three tests were also conducted to investigate the retention of balance learning ability. Data were analyzed using the IBM Statistical Package for the Social Sciences (SPSS) 23.00 with a significance level α = 0.05.

## 3. Results

In the experimental group, 1 participant did not perform the measurement to maintain learning in the retention phase. In the 1st control group, 1 participant performed only the 1st measurement at the beginning of the study. In the 2nd control group, 2 participants performed only the 1st measurement, and 1 participant performed the 1st and the 2nd measurements due to injury.

No statistically significant differences were found between the three groups in age, height, weight, BMI, and number of sprains. Descriptive statistics of the (a) Y-Balance test, (b) center of pressure distribution length, (c) center of pressure distribution velocity, and (d) CAIT at the beginning, end, and retention phase between the three groups are presented in Table 2.

### 3.1. Examining the Comparisons and Retention of Balance Learning with Y-Balance Test

The Repeated Measures ANOVA (mixed ANOVA) examined the differences between the three different groups (between-subjects effect) and between the three repeated measurements (within-subjects effect). The results revealed only a significant main effect for intervention between the three repeated measurements (within-subjects effect) (F(2,36) =5.96, *p* = 0.006, partial η^2^ = 0.249), with no significant interaction effect between the three different groups. Additionally, the between-subjects effects (i.e., between-subject factor) were not statistically significant to perform post hoc analyses.

In order to further examine the within-subjects effects and assess the retention of balance learning ability, three paired samples *t*-tests with a Bonferroni adjustment were conducted (significance level for multiple comparisons *p* = 0.017), comparing the three measurements in each group (1st and 2nd measurement, 1st and 3rd measurement, 2nd with 3rd measurement). The results of the paired samples *t*-tests showed that the intervention group with external verbal feedback demonstrated a statistically significant difference between the 1st and the 2nd measurement (t = −3.44, *p* = 0.040), between the 1st and the 3rd measurement (t = −2.93, *p* = 0.010), but not between the 2nd and 3rd measurement (t = −0.31, *p* = 0.380).

The paired samples *t*-test for the 1st control group without feedback showed a tendency towards statistical significance between the 1st and 2nd measurement (t = −2.36, *p* = 0.020), between the 1st and 3rd measurement (t = −2.58, *p* = 0.021), but not between the 2nd and the 3rd measurement (t = −1.68, *p* = 0.071). In contrast, the paired samples *t*-test for the 2nd control group without balance training and without feedback showed no statistically significant difference between the three measurements.

These results indicate that only the experimental group with external verbal feedback demonstrated maintenance of dynamic balance learning ability during the retention phase of the dynamic ability learning process.

### 3.2. Examining the Comparisons and Retention of Balance on Center Length and Center Velocity Pressure Distribution during the Y-Balance Test

The Repeated Measures ANOVA (mixed ANOVA) examined the differences between the three different groups (between-subjects effect) and between the three repeated measurements (within-subjects effect). However, the results showed no statistically significant effects, neither between the three groups nor between the three repeated measurements.

Regarding the retention of balance learning ability on center length and center velocity pressure distribution, the test of normal distribution of variables using the Shapiro-Wilk index indicated the need for using non-parametric analysis. Therefore, the Kruskal-Wallis non-parametric H test comparisons were conducted. However, the results showed non-statistically significant differences for the balance ability and the learning retention test for the 2nd and 3rd measurements of the center length and the velocity pressure distribution among the three groups.

### 3.3. Examining the Comparisons and Retention of Learning of Balance on the CAIT

The Repeated Measures ANOVA (mixed ANOVA) examined differences between the three different groups (between-subjects effect) and between the three repeated measures (within-subjects effect). The results revealed a statistically significant main effect between the three repeated measures (within-subjects effect) with Greenhouse-Geisser correction (F (2,36) = 9.33, *p* = 0.005, partial η^2^ = 0.342) and a significant interaction effect between the three measurements and the group with a Greenhouse-Geisser (F(4,36) = 3.18, *p* = 0.058, partial η^2^ = 0.261). Consequently, all three groups, including those with no balance training, demonstrated significant overall improvement in their balance on the CAI. However, the between-subjects effects (i.e., between-subject factor) were not statistically significant to perform post hoc analyses. Therefore, external verbal feedback did not show a positive impact on the balance ability among the three different groups.

To further examine the within-subjects effects and the retention of balance learning ability, three paired samples tests with a Bonferroni adjustment were conducted (significance level for the multiple comparisons *p* = 0.017), comparing the three measurements in each group (1st and 2nd measurement, 1st and 3rd measurement, 2nd and 3rd measurement). The results of the paired *t*-tests showed that the intervention group with external verbal feedback demonstrated a statistically significant difference between the 1st and the 2nd measurement (t = −3.12, *p* = 0.006), between the 1st and 3rd measurement (t = −3.32, *p* = 0.005), but not between the 2nd and 3rd measurement (t = −1.00, *p* = 0.173), indicating maintenance of dynamic balance learning ability in the experimental group with external verbal feedback. The paired *t*-tests for the 1st control group without feedback and the 2nd control group without balance training and without feedback showed no statistically significant differences between the three measurements.

## 4. Discussion

The results indicate that balance training positively affects the dynamic balance of individuals with CAI. Additionally, external verbal feedback significantly contributes to the sustained retention of balance learning ability in individuals with CAI. However, there was no observed positive impact of external verbal feedback on the balance ability of individuals with CAI compared to those who did not receive feedback during the balance training. Therefore, larger sample sizes are necessary to confirm these findings.

Only one other study has investigated the effectiveness of feedback on the balance ability of participants with CAI. Jaffri and Saliba [33] utilized the Star Excursion Balance Test with and without verbal feedback, reporting improved balance ability after feedback. However, their study did not show any improvements in the length and velocity of the center of pressure distribution, which are crucial indicators of instability. These parameters are important because participants may exhibit good performance in the Star Excursion Balance Test but still have greater oscillation of the pressure center and velocity, indicating instability. Furthermore, Jaffri and Saliba did not administer the Cumberland Ankle Instability Tool (CAIT), thus they did not assess whether there was an improvement in instability symptoms before and after feedback. In contrast, our study demonstrated an improvement in ankle joint instability symptoms at the end of the intervention program compared to the beginning. Additionally, real-time external feedback using a crossline laser device did not result in changes in neuromechanical characteristics in the entire lower extremity (i.e., ankle, knee, and hip joints) during static postural control between feedback and no feedback conditions in 18 individuals with CAI [49].

In the cognitive phase of motor skill acquisition, where participants follow instructions during the initial learning stage of a skill, verbal feedback emerges as a highly effective technique. During this phase, participants require more feedback than in other stages of motor skill learning. Consequently, participants demonstrated improvement in their motor skills, especially in balance, following these feedback instructions [50].

The improvements observed following the balance program, facilitated by feedback, may be attributed to a reduction in fear, leading to increased confidence in participants’ performance. It seems that a psychological mechanism could be influencing the relationship between feedback and balance ability. While the reduced performance in the dynamic balance test in CAI is attributed to a reduced trajectory range, balance deficits may also be associated with psychological limitations stemming from the chronic pathology. Ohno et al. [51] found a positive correlation between stress levels and the center of balance in participants with CAI. Feedback may increase motivation, focus on motor function, enhance activity in the athlete’s higher brain centers, and familiarize the cerebral cortex by reducing psychological barriers [33]. Further research is warranted to elucidate the relationship between feedback, psychological factors, and balance improvement, particularly in the context of CAI. If a relationship is identified, further investigation is needed to discern the specific mechanisms accountable for the observed enhancement in balance ability.

Rendos et al. [34] investigated the maintenance of gait learning in patients with post-stroke hemiparesis using functional electrical stimulation and feedback after 24 h. The combination of these techniques improved gait ability. Their study had a smaller sample size than the present study, which included an intervention group but lacked a control group. Additionally, while Rendos et al. assessed retention 24 h after the intervention, our study had a longer learning retention interval. We extended the abstinence period to 4 weeks to minimize the impact of performance variables. This approach aimed to ensure that participants’ performance on each test primarily reflected the effectiveness of the taught and trained balance training, as previously demonstrated [27].

The sex of participants may influence the outcomes of the current study. Previous research suggests that female athletes in basketball and soccer often demonstrate superior single-legged balance abilities compared to their male counterparts. However, female basketball athletes may exhibit increased ankle range of eversion and inversion, indicating potential greater functional instability in the ankle joint [52]. Ottaviani et al. [53] observed that, on average, young women have 39% less eversion muscle strength than young men. These findings underscore the importance of considering sex-specific factors when interpreting and generalizing the results of the present study.

Moreover, male basketball athletes often exhibit higher vertical jump and sprint run performance compared to their female counterparts, indicating better functional performance measures. However, this sex difference becomes non-significant when ankle strengths are normalized by body size. In contrast, Greene et al. [54] found no significant differences between male and female basketball athletes in a single-limb balance time test. Additionally, no significant sex differences were reported for ankle range of motion in plantar flexion and dorsiflexion. These insights underscore the importance of considering sex-specific factors when interpreting and generalizing the results of the present study.

The current study has several notable strengths. Firstly, the inclusion of two control groups enhances the validity of the results, providing a more robust basis for drawing conclusions. Secondly, the utilization of electronic equipment for assessing improvements in instability and center of pressure distribution length and velocity adds precision to the measurements. A third strength lies in the investigation of balance maintenance post-intervention, shedding light on the endurance of balance improvements over time. Moreover, the study holds significance as the first to explore the impact of external verbal feedback on the shift of the center of pressure and velocity within a balance program. This novel aspect contributes valuable insights to the existing body of knowledge. Additionally, the study breaks new ground by examining the effects of feedback in conjunction with the Y balance test, further expanding the understanding of feedback in balance assessments. Lastly, the examination of balance learning maintenance after a 4-week abstinence period sets this study apart, filling a gap in the existing literature.

### Limitations and Future Recommendations

The present study is subject to several limitations. The primary constraint lies in the small and diverse sample size, comprising both male and female participants engaged in various sports. While it was challenging to find athletes with identical demographic characteristics and injury types, this heterogeneity may affect the generalizability of the results. Increasing the sample size could potentially unveil additional positive effects of feedback on balance. Another limitation is the incomplete assessment of learning retention due to injuries during the study, reducing the sample size for this analysis to *n* = 30. Additionally, the study’s findings may not be generalized beyond athletes or those with a similar chronic musculoskeletal injury. Future research should aim for larger, homogeneous samples in terms of gender and sport to overcome these limitations and enhance the generalizability of the results.

To address these limitations, future studies should explore the effects of different balance programs with feedback on athletes sharing similar musculoskeletal injuries. Combining strength training with balance programs and incorporating additional motor learning techniques, such as attention, could offer a more comprehensive approach to rehabilitation and performance enhancement. Subjective evaluations should extend beyond balance to include neuromuscular coordination and proprioception, providing a more holistic understanding of athletes’ motor abilities. New experimental designs, considering the complementary application of various motor learning and control methods, are proposed to mitigate limitations and provide more robust evidence.

Additionally, there is a crucial need for investigations into the psychophysiological processes of motor learning techniques that unfold during sports injury rehabilitation. Understanding these processes could inform the development of more effective rehabilitation protocols tailored to individual athlete needs.

Moreover, future studies should examine the balance variable not only on the leg with CAI but also on the contralateral “healthy” leg. This approach is essential due to the “bilateral consequences of unilateral injury” hypothesis, which suggests that unilateral injury could lead to bilateral consequences, resulting in altered postural control on the “healthy” limb due to a general reorganization of the sensorimotor system [55]. By considering both limbs, researchers can gain a more comprehensive understanding of the effects of injury on balance and motor control.

Furthermore, future research should investigate the impact of feedback on other joints, such as the knee or shoulder, with different sports injuries (e.g., shoulder impingement syndrome, patellofemoral pain syndrome). This comprehensive approach would contribute to a more nuanced understanding of the effects of feedback across various musculoskeletal conditions.

## 5. Conclusions

Balance training has a positive effect on the dynamic balance of the CAI. Although external verbal feedback did not have any observed positive impact on the balance ability of individuals with CAI compared to those who did not receive feedback during the balance training, it contributes to the sustained retention of balance learning ability even after the completion of the balance and feedback intervention. Replicating the present study with a larger and more homogeneous sample is imperative to validate the reliability of these results. By expanding our understanding of the role of feedback in improving balance among individuals with CAI, we can potentially advance rehabilitation strategies and optimize the recovery process for sports-related injuries.

## Figures and Tables

**Table 1:** Demographic data (mean ± SD).

	Age (Years) (Μ ± SD)	Height (cm) (Μ ± SD)	Weight (Kilogram) (Μ ± SD)	BMI (kg/m^2^) (M ± SD)	Time since the Last Sprain (Months) (Μ ± SD)	History of Sprains (Number) (Μ ± SD)
Experimental group with feedback	19.8 ± 1.03	172.3 ± 9.31	69.10 ± 11.78	23.35± 10.24	12.6 ± 7.56	2.4 ± 1.28
1st Control group without feedback	21.4 ± 1.95	172.9 ± 10.16	70.80± 10.59	23.93 ± 10.23	6.2 ±4.02	2.9 ± 1.13
2nd Control group without balance training-without feedback	23.7 ± 1.63	181.8 ± 4.96	80.10 ± 7.78	24.58 ± 6.34	11.5 ± 7.89	2.6 ± 1.35

**Table 2:** Descriptive statistics of the Y-Balance Test, of the center of pressure distribution length and velocity, CAIT (mean ± SD).

	Experimental Group with Feedback			1st Control Group without Feedback			2nd Control Group without Balance Training—without Feedback		
Variables	1st measurement	2nd measurement	3nd measurement	1st measurement	2nd measurement	3nd measurement	1st measurement	2nd measurement	3nd measurement
Y-Balance	96.13 ± 9.32	99.97 ± 7.38	100.19 ± 6.18	96.13 ± 9.32	99.97 ± 7.38	100.19 ± 6.18	94.32 ± 5.99	93.71 ± 7.49	95.18 ± 5.68
Centre length pressure distribution	567.61 ± 168.7	514.51 ± 176.26	524.44 ± 167.1	659.64 ± 205.8	671.99 ± 167.3	588.46 ± 119.7	656.47 ± 211.7	624.22 ± 159.7	609.7± `186.7
Pressure distribution centre velocity	56.75 ± 16.87	51.44 ± 17.62	52.44 ± 16.42	56.75 ± 16.87	51.44 ± 17.62	52.44 ± 16.42	56.75 ± 16.87	51.44 ± 17.62	52.44 ± 16.42
CAIT	17.40 ± 4.88	21.90 ± 1.85	22 ± 1.73	19.80 ± 3.12	22.67 ± 1.5	22.11 ± 1.76	20.60 ± 3.02	20.88 ± 2.58	20.86 ± 2.79

Abbreviation: CAIT, Cumberland Ankle Instability Tool.

## Data Availability

Data available upon reasonable request.
